# Combined fMRI-MRS acquires simultaneous glutamate and BOLD-fMRI signals in the human brain

**DOI:** 10.1016/j.neuroimage.2017.04.030

**Published:** 2017-07-15

**Authors:** I. Betina Ip, Adam Berrington, Aaron T. Hess, Andrew J. Parker, Uzay E. Emir, Holly Bridge

**Affiliations:** a Department of Physiology, Anatomy & Genetics, University of Oxford, Oxfordshire OX1 3PT, UK; b Oxford Centre for Functional MRI of the Brain (FMRIB), University of Oxford, Oxford, Oxfordshire OX3 9DU, UK; c Centre for Clinical Magnetic Resonance Research (OCMR), University of Oxford, Oxford, Oxfordshire OX3 9DU, UK

**Keywords:** Functional Spectroscopy, BOLD fMRI, Glutamate, Neurochemistry, Visual cortex

## Abstract

Combined fMRI-MRS is a novel method to non-invasively investigate functional activation in the human brain using simultaneous acquisition of hemodynamic and neurochemical measures. The aim of the current study was to quantify neural activity using combined fMRI-MRS at 7 T. BOLD-fMRI and semi-LASER localization MRS data were acquired from the visual cortex of 13 participants during short blocks (64 s) of flickering checkerboards. We demonstrate a correlation between glutamate and BOLD-fMRI time courses (R=0.381, p=0.031). In addition, we show increases in BOLD-fMRI (1.43±0.17%) and glutamate concentrations (0.15±0.05 I.U., ~2%) during visual stimulation. In contrast, we observed no change in glutamate concentrations in resting state MRS data during sham stimulation periods. Spectral line width changes generated by the BOLD-response were corrected using line broadening. In summary, our results establish the feasibility of concurrent measurements of BOLD-fMRI and neurochemicals using a novel combined fMRI-MRS sequence. Our findings strengthen the link between glutamate and functional activity in the human brain by demonstrating a significant correlation of BOLD-fMRI and glutamate over time, and by showing ~2% glutamate increases during 64 s of visual stimulation. Our tool may become useful for studies characterizing functional dynamics between neurochemicals and hemodynamics in health and disease.

## Highlights

• Novel MRI sequence measures hemodynamics and neurochemistry in same TR.
• Stimulation block duration relevant for functional experiments (64s).
• BOLD-fMRI and glutamate time courses correlate during functional stimulation.
• Visual stimulation increases glutamate concentrations.
• Useful to study fundamental relationship between hemodynamics and neurochemistry.

## Graphical abstract

**Figure:**
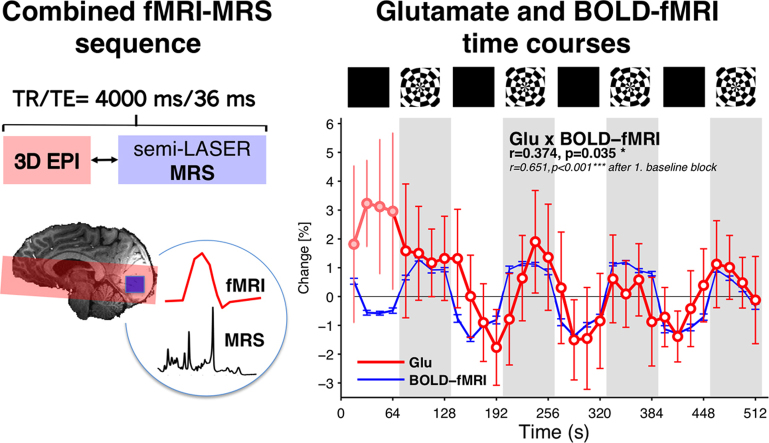

## Introduction

The blood-oxygenation level dependent (BOLD)-fMRI response is one of the most widely used measures of neural activity (Ogawa et al., 1990) yet is not a direct measure of action potentials, or synaptic activity. BOLD-fMRI reflects a spectrum of energy and blood-flow dependent processes (Logothetis et al., 2001) which are not fully understood (Logothetis, 2008, Hall et al., 2016). ^1^H-MRS is a non-invasive measure of absolute concentrations of neurochemicals and, particularly in the absence of any sensory stimulation, has been exploited to identify biomarkers of normal and pathological brain states (Oz et al., 2014). While several recent studies have measured functional ^1^H-MRS during specific tasks (Mangia et al., 2006, Mangia et al., 2007, Lin et al., 2012, Schaller et al., 2013, Schaller et al., 2014, Apsvalka et al., 2015, Bednarik et al., 2015), no study to date has quantified simultaneous changes in neurochemicals and brain activity using BOLD-fMRI. Here, we provide the first demonstration of combined fMRI-MRS measurements, and reveal a specific relationship between changes in BOLD-fMRI and glutamate at time scales relevant to conventional fMRI block design experiments (64 s). These results cannot be explained either by line narrowing during BOLD-changes (Zhu and Chen, 2001) or resting state variations in glutamate.

## Materials and methods

### Participants

Eighteen volunteers (9 females, mean age 28.71±5.62 years), including two of the authors, were recruited for the main study. All had normal, or corrected-to-normal, vision and normal stereo-acuity (< 120 arc sec, TNO Stereo test, Lameris, Utrecht). Five participants were excluded from analysis due to one or several of the following reasons: early termination of experiment; difficult MRS voxel placement as evidenced by negative BOLD-fMRI signal and/or poor signal-to-noise in the metabolite spectra. The final data set was composed of 13 subjects (7 females). Each volunteer took part in one behavioral session to assess their vision, and one MRI session. Volunteers received a reimbursement of £10 for the behavioral testing session and £25 for the MRI session. All gave informed written consent, approved by the University of Oxford Research Ethics Committee (MSD-IDREC-C1-2014-146).

### MR protocol

MR data were collected using a 7 T whole body MR-scanner (Siemens, Erlangen) with a Nova Medical head coil (single transmit, 32 receive channels). Anatomical images were collected with a 1-mm isotropic resolution (MPRAGE, repetition time TR=2.2 s, inversion time T_I_=1.05 s, echo time TE=2.82 ms, FOV=192×192×176 mm, flip angle=7°, total acquisition time=171 s) for the placement of the visual cortex voxel-of-interest (VOI). A 2×2×2 cm MRS VOI was positioned in the occipital lobe, centered along the midline and the calcarine sulcus.

**Fig. 1:**
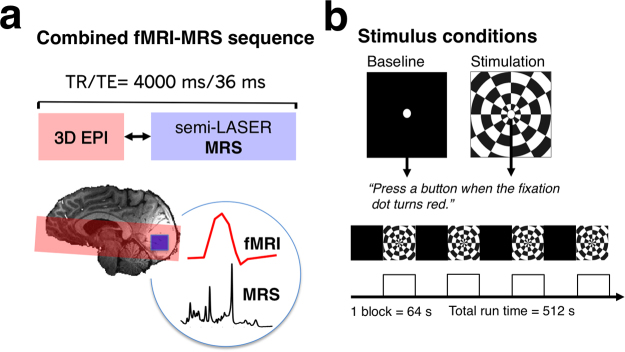
(a) Diagram shows the MR sequence consisting of a 3D BOLD echo planar imaging (3D-EPI) and semi-LASER MR-spectroscopy sequence in the same TR, with diagram below illustrating the 2×2×2 cm MRS voxel in the occipital lobe (blue square) and the EPI slice coverage (red outline), overlaid on a high resolution anatomical image. (b) Experimental design showing stimulus conditions, consisting of a baseline (black screen, 64 s) and a flashing checkerboard (64 s). Each participant took part in a single functional MRS visual stimulation experiment, consisting of four cycles of baseline and stimulation blocks. Subjects performed a fixation task throughout the 8 min 24 s experiment.

Fig. 1a shows a diagram of the combined fMRI-MRS sequence, based on a sequence developed by Hess et al. (2011). In the same TR of 4 s, BOLD-fMRI (3D EPI, resolution=4.3×4.3×4.3 mm; flip angle=5°, repetition time TR_epi_=40 ms, TE=25 ms, FOV=240 mm, 16 slices) and MRS data were acquired. MRS data were acquired using short-echo semi-localisation by adiabatic selective refocusing (semi-LASER) pulse sequence (TE=36 ms, TR_mrs_=4 s) with VAPOR water suppression and outer volume suppression (Oz and Tkac, 2011, van de Bank et al., 2015). Semi-LASER sequences have a high test-retest reliability at 7 T (Terpstra et al., 2015) and minimal chemical shift displacement at ultra-high field MR imaging. A delay between fMRI and MRS acquisition (250 ms) was inserted to minimize potential eddy current effects from the EPI read-out (Hess et al., 2011).

### Experimental design

Stimuli were generated on a MacBook Pro laptop using Psychtoolbox-3 (Brainard, 1997) and custom written Matlab scripts. An Eiki LC-XL 100 projector (1028×768 pixels, 60 Hz) displayed the images onto a back-projection screen (DA-LITE Milestone AV Technologies, Minnesota). For each subject, a resting state experiment (‘eyes shut’) was collected during which no visual stimulation was delivered, and subjects were instructed to keep their eyes shut for the entire scan. During visual stimulation, subjects viewed the screen using an angled mirror, mounted on the head-coil (viewing distance=60 cm). Fig. 1b shows a diagram of the visual stimuli and experimental protocol. Visual stimulation consisted of a baseline-stimulation block design (stimulus size=19.82°×14.25°, block length=64 s, number of cycles=4). During the stimulation period a full-field contrast-reversing checkerboard was presented (8 Hz flicker, mean luminance=385 cd/m^2^; 50% contrast, 64 s duration). The baseline period was a uniform black screen (2.33 cd/m^2^; 64 s duration). A white central fixation dot (0.5°) was visible at all times, and subjects pressed a button on a button-box when it randomly turned red (500 ms) about once every three seconds. The purpose of the task was to encourage central fixation and steady levels of attention. The type of visual stimulation used is known to target the visual cortex and is unlikely to generalize across cortical regions. In support, a previous study using similar checkerboard stimuli in a prolonged stimulation design has found no changes in metabolites outside of the visual cortex (Mangia et al., 2006). Each participant took part in two combined fMRI-MRS experiments: The resting state scan was collected first (128 averages, ‘eyes closed no stimulation’), followed by the functional scan (128 averages, ‘four cycles of flashing checkerboards and baseline’). Supporting scans for anatomical registration were collected last.

### fMRI analysis

fMRI data analysis was performed using FEAT (FMRI Expert Analysis Tool) v. 6.00, part of the FSL software distribution (FMRIB's Software Library, www.fmrib.ox.ac.uk/fsl). Data were pre-processed using motion correction MCFLIRT (Jenkinson et al., 2002); non-brain tissue extraction (Smith, 2002); spatial smoothing using Gaussian kernel of FWHM=5 mm, grand-mean intensity normalization and high pass temporal filtering (Gaussian-weighted least squares straight line fitting, main experiment=132 s; resting state data=250 s). Functional images were registered to an initial 2-mm structural image (6 DOF), and then to the 1-mm isotropic T1-weighted structural image using boundary-based registration (BBR) in FLIRT (Jenkinson and Smith, 2001, Jenkinson et al., 2002). Percentage BOLD-change in the MRS-voxel was calculated using Featquery. The group activation map was calculated using FLAME (FMRIB's Local Analysis of Mixed Effects), with z-statistic threshold>2.3 and cluster-correction threshold of p<0.05. Participants maintained very steady head position throughout the scan, as indicated by motion estimates from MCFLIRT (Jenkinson et al., 2002). Absolute motion displacement referenced to the center of slice was 0.228±0.056 mm (mean N=13, ±std). Relative motion displacement referenced to the preceding time point was 0.173±0.056 mm.

### Dielectric pad

A dielectric pad measuring 110×110×5 mm^3^ containing a suspension of Barium Titanate (BaTiO_3)_ and deuterated water (mass-mass ratio of 3:1) was placed behind the occiput of each subject to increase the extent of the effective transmit field (Luo et al., 2013, Lemke et al., 2015). The pad was positioned so that the center of the bias field was symmetric with the midline. Dielectric pads can increase transmit field efficiency (>100%) in regions close to the pad without affecting specific absorption rate and B_0_ field homogeneity (Teeuwisse et al., 2012).

### Data reduction

Previous functional MRS studies have excluded up to 50% of data to obtain stable metabolite measurements, by using the second half of prolonged visual stimulation blocks (Mangia et al., 2006, Mangia et al., 2007, Schaller et al., 2013, Bednarik et al., 2015); or focused on specific experimental cycles (Just et al., 2013). We excluded the first two time averages (2 TR=8 s) of each block, under the assumption that metabolite spectra are unstable during the period where the BOLD-amplitude is known to peak (Buxton et al., 2004). Excluding the first two TRs from every block is equivalent to excluding 12.5% of the data.

### Metabolite quantification

MRS data were eddy-current corrected using the unsuppressed water signal from the same ROI, frequency aligned to the tNAA singlet at 2.01 ppm, and phase corrected using a least-square algorithm using MRspa (https://www.cmrr.umn.edu/downloads/mrspa/), a semi-automated matlab routine. Preprocessed data were then analyzed using LCModel (Provencher, 1993, Provencher, 2001). Spectra were of good quality in all participants (see Suppl. Materials Fig. S1). Metabolite concentrations were estimated using a basis set of alanine (Ala); ascorbate/vitamin C (Asc); aspartate (Asp); glycerophosphorylcholine (GPC); phosphorylcholine (PCho); creatine (Cr); phosphocreatine (PCr); γ-aminobutyricacid (GABA); glucose (Glc); glutamine (Gln); glutamate (Glu); glutathione (GSH); inositol (Ins); lactate (Lac); phosphoethanolamine (PE); scyllo-inositol (sIns); taurine (Tau); N-acetyl-aspartate multiplet (mNAA); N-acetyl-aspartate singlet (sNAA); Acetyl moiety of N-acetylaspartylglutamate (sNAAG); Aspartyl moiety of NAAG (mNAAG); Glutamate moiety of NAAG (gNAAG).

We followed the same macromolecule inclusion procedure as Bednarik et al. (2015). Macromolecular spectra were included in the LCModel basis set. Macromolecule spectra acquired from the occipital cortex from 3 healthy volunteers, using an inversion recovery sequence (TR=3 s, TE=36 ms, inversion time TI=0.685 s), were included in the model spectra. The residual signal of the methylene of tCr at 3.93 ppm was removed by post processing and the high-frequency noise was suppressed using a Gaussian filter (σ=0.05 s) before including the macromolecule spectrum into the LCModel basis set. Finally, no changes in macromolecules were observed when comparing baseline and stimulation concentrations of glutamate.

The percentage of cerebro-spinal fluid in the MRS voxel was determined using automated tissue segmentation (FSL v6.0 FAST (Zhang et al., 2001)) and custom written scripts. Metabolites were quantified in institutional units (I.U.) relative to the unsuppressed water collected from the same VOI (Provencher, 2013). Estimated metabolite concentrations were only corrected for the amount of cerebro-spinal fluid (CSF), no corrections were performed for T1 and T2 relaxations. The average percentage of CSF in the MRS voxel was 8.16±3.58% (N=13,±std). Metabolite estimates with a standard deviation of Cramèr-Rao Lower Bounds (CRLB) <20% were considered reliable. Metabolites with CRLB > 20% were considered too weak to be reliable (alanine, aspartate, GPC, GABA, glucose, lactate, mNAAG, gNAAG). These metabolites were estimated in LCModel analysis but not considered in further analysis. The concentrations sum of the pair were reported for GPC+PCho (total Choline, tCho) and PCR+Cr (total Creatine, tCr).

### Difference spectrum

Before data analysis, the first two averages (2 TR=8 s) at the start of each baseline and stimulation block were removed. Individual subjects’ spectra were manually aligned to the singlet of N-acetyl aspartate (NAA) at 2.01 ppm of a reference subject. Spectra were then summed across subjects (14 averages×4 blocks×13 participants=728 spectra/condition). The single subject spectrum acquired during stimulation was line broadened to match the width of the tCr during the baseline spectrum before summing. The difference spectrum is a subtraction of the average spectrum acquired during baseline from the average stimulation spectrum.

### Time course calculation

To calculate the time course of metabolite concentrations within subject, four consecutive averages were summed into one spectrum and analyzed using LCModel, yielding a time course composed of 32 data points and a temporal resolution of 16 s. Before correlation analysis, single subject glutamate time courses were smoothed using a temporal filter with a 3-point moving average. Glutamate changes were quantified as percentage change from the average baseline concentration, using the equation:

$$\mathrm{\Delta Glutamate}\;{\;=\;((G}_{i}–\;{\text{G}}_{\mathrm{baseline}}{)\;/\;\text{G}}_{\mathrm{baseline}})*100$$

Where G_i_=glutamate concentration at time point i, G _baseline_=average baseline glutmate concentration. A positive change represents an increase in glutamate concentration over baseline, and a negative change a decrease. Single subject time courses are presented in Supplementary Materials Fig. S2.

## Results

**Fig. 2:**
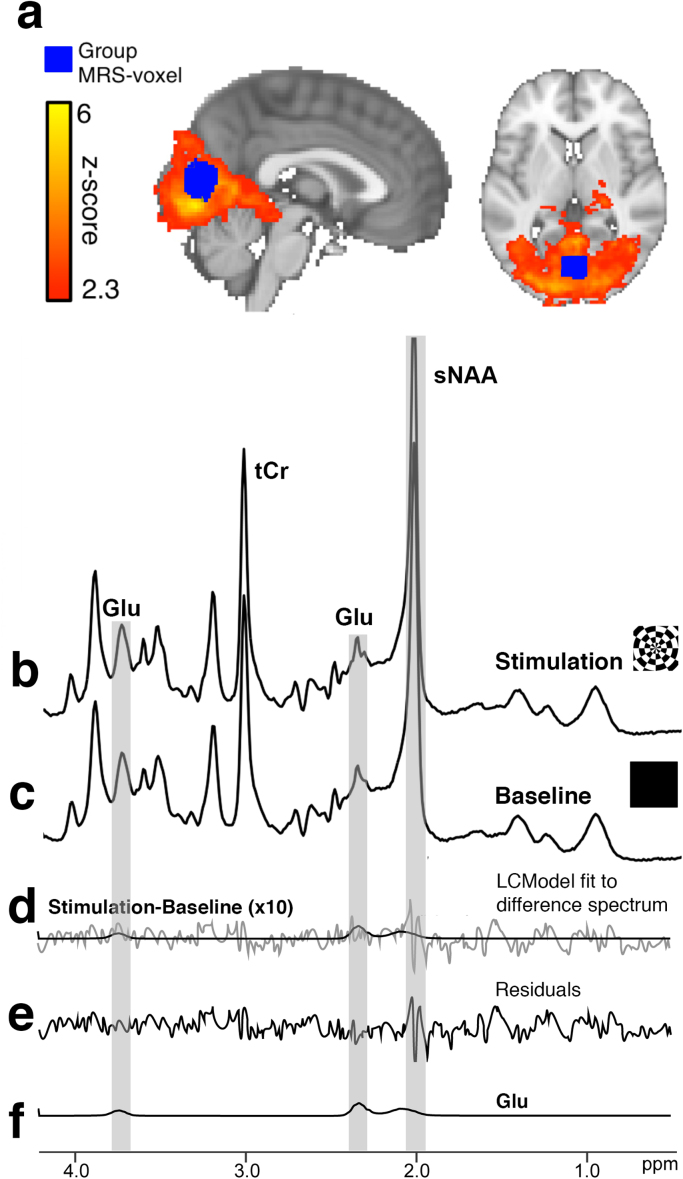
(a) Red heat map shows thresholded group activation generated by the comparison of stimulation vs. baseline (z-stat >2.3, cluster-corrected at p<0.05) overlaid on sagittal (left) and horizontal (right) standard brain sections. Blue colour map shows region where >50% of the group MRS-voxel overlapped (N=13). Combined MRS data during stimulation (b) and baseline (c) conditions. Each baseline and stimulation spectra are composed of the sum of 13 subjects×4 repetitions×14 spectra/block=728 total spectra/condition. (d) Difference spectrum, created by subtracting baseline from stimulation with LCModel fit. (e) Residual of LCModel fit of the difference spectrum. (f) LCModel fit of glutamate on the difference spectrum. Pale vertical gray lines indicate the singlet positions Glutamate and singlet of N-acetylaspartate (NAA) at 2.01 ppm. Spectra in b-c are plotted to the same y-axis scale; d-f have been plotted on an expanded vertical scale.

We developed and implemented a novel functional MR-sequence that simultaneously recorded BOLD-fMRI and ^1^H-MRS (combined fMRI-MRS). 3D-EPI and semi-LASER spectroscopy data were acquired in the same 4 s TR from a visual cortex voxel, while participants viewed visual stimulation consisting of four alternations of a baseline black screen (64 s; ‘baseline’) followed by presentation of a flickering (8 Hz) checkerboard (64 s; ‘stimulation’). Visual stimulation resulted in significant increases of BOLD-fMRI amplitude inside the MRS-VOI (1.43±0.17%, N=13, mean±s.e.m; *t*-test: t(12)=8.24, p<0.001). The overlap between the mean position of the MRS-voxel and activation generated by visual stimulation is shown in Fig. 2a. Visual stimulation activated 90.97±2.66% (N=13, mean±s.e.m) of voxels inside the MRS-VOI.

Simultaneously acquired MRS spectra provided reliable estimates of multiple metabolites with Cramèr-Rao Lower Bounds (CRLB)<20%. To compare stimulation and baseline MRS spectra, spectroscopy data across participants were summed into a stimulation (Fig. 2b) and baseline (Fig. 2c) spectrum, each composed of 728 averages. A difference spectrum (Fig. 2d), generated by subtracting baseline from stimulation spectrum, reveals peaks at the position of glutamate, which are modeled by LCModel (Fig. 2f). The residuals of the LCModel fit are presented in Fig. 2e.

**Fig. 3:**
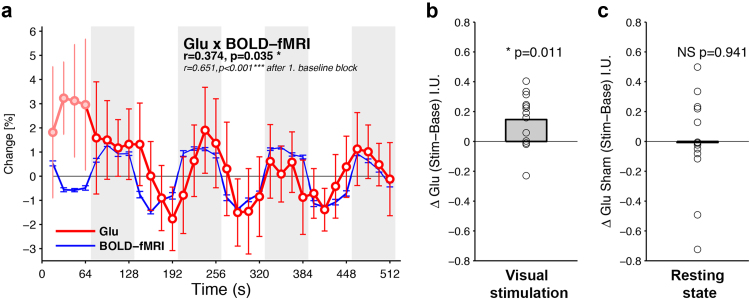
(a) Glutamate response over time, as a percentage of average baseline glutamate concentration. Data represent the responses to a single 512 s stimulation experiment collected per subject, averaged across subjects (N=13, ±s.e.m). Pale red line and filled circles indicates transient response in the first baseline period. BOLD-fMRI response is shown in blue (N=13, ±s.e.m). R- and p-values in bold font reflect Pearson's Correlation using data from the full time course. Italicized R- and p-values are Pearson's Correlation using data after the first baseline period. The baseline periods are indicated as white and stimulus periods as gray shaded background. (b) Bar graph shows ΔGlu (Stim-Base). (c) Bar graph shows resting state ΔGlu analyzed as if stimulation had been delivered (‘Sham’). During the resting state scan, participants kept their eyes shut and no stimulation was delivered (N=13, ±s.e.m). P-value indicates result of a one-sample *T*-test. NS=not significant, *=p <0.05.

To investigate the relationship between glutamate and the BOLD-fMRI response over time, data were averaged across every four averages within subject (1 data point: resolution=16 s, mean±std, CRLB=8.438±0.90%), smoothed with a 3 point moving average and then averaged across 13 subjects (Fig. 3a, red line). BOLD-fMRI responses within the MRS voxel were averaged in the same way, z-normalized and plotted alongside the glutamate response (Fig. 3a, blue line). Glutamate was significantly correlated with the BOLD-response across the scan (r(31)=0.381, p=0.031). This correlation remained after line broadening of spectra collected during stimulation periods (r(31)=0.374, p=0.035). Glutamate during the first baseline period (Fig. 3a, filled circles) may reflect a familiarization period, and correlation increased if this period was removed (r(27)=0.631, p<0.001). To determine whether any correlations could be explained by resting state glutamate, we correlated the resting state glutamate signal with BOLD-fMRI data from the visual stimulation experiment. No significant correlations were observed (r(31)=0.162, p=0.374; r(27)=0.156, p=0.429). In addition, we found no correlation in the resting state scan between glutamate and the BOLD-fMRI time course (r(31)=0.065, p=0.724; r(27)=0.11, p=0.572).

To measure metabolite changes time locked to stimulation and baseline periods, separate LCModel analyses were performed for each condition. First, metabolite spectra collected during baseline or stimulation periods were summed within subject and quantified using LCModel. The resulting metabolite concentration estimates were averaged across 13 subjects. Baseline concentrations were then subtracted from stimulation concentrations to obtain a metric of relative change in units ΔGlu I.U. The absolute glutamate concentration increased by 0.15±0.05 I.U. (Fig. 3b, one-sample *t*-test: t(12)=−2.98, p=0.011) during stimulation, equivalent to an increase of 1.92±0.66% from the baseline concentration. This increase remained significant also after applying line broadening to stimulation spectra to account for the BOLD-effect (1.65±0.59%, p=0.014). No other metabolite response was significant before and after BOLD-correction. To assess the relationship between glutamate and BOLD-fMRI when the same region of cortex was not stimulated, resting state data were analyzed as if stimulation had been delivered (‘sham’ stimulation). During resting state scans, subjects had their eyes closed and no stimulation was delivered. No increase in resting state glutamate was observed (one-sample *t*-test: t(12)=0.27, p=0.941, see Fig. 3c). Resting state glutamate responses were more variable than during visual stimulation. This was likely due to the fact that visual cortex responses were not constrained by an attention demanding visual task and stimulation.

In functional activation paradigms using MR spectroscopy, the BOLD-signal causes line narrowing of MR spectra (Zhu and Chen, 2001). If left uncorrected, spectral line narrowing results in an apparent increase in absolute metabolite concentration (Mangia et al., 2006, Bednarik et al., 2015) for a number of metabolites. We identified line narrowing in the MR spectra using the tCr singlet peak at 3.03 ppm (Zhu and Chen, 2001).

Under the assumption that tCr remains stable during brain activation, any changes in line width are considered BOLD induced. Automated fitting of Lorentzian functions to the singlet of tCr revealed narrowing of spectral line widths during stimulation periods. tCr line widths narrowed by 2% during stimulation compared to baseline (N=13; baseline: 9.72±0.85 Hz; stimulation: 9.51±0.82 Hz, paired *t*-test, p<0.001). In addition, change in tCr line width was correlated with BOLD-change (N=13, R=0.612, p=0.026).

## Discussion

Our work presents the first opportunity to directly link hemodynamic and neurochemical responses in the human brain using a novel combined fMRI-MRS sequence. As a proof-of-concept, we stimulated the human visual cortex using blocks of 64 s flickering checkerboard. Our results show a strong link between BOLD-responses and glutamate: (i) average BOLD signal and glutamate concentration increased during stimulation and (ii) glutamate and BOLD-fMRI signals correlated significantly over time. We show that the relationship between glutamate and the BOLD response is specific to the activated visual cortex, and absent in the resting visual cortex.

Previous studies have shown changes in glutamate on the order of 2–4% (Mangia et al., 2006, Lin et al., 2012, Schaller et al., 2013, Bednarik et al., 2015) after several minutes of visual stimulation. We show that a change of ~2% can be generated with a substantially shorter stimulation times. In addition, we strengthen the link between glutamate and the hemodynamic response by demonstrating that glutamate and BOLD-fMRI response correlate over time. Previous studies have shown increases in lactate, and decreases in aspartate. Our data did not permit measurement of lactate and aspartate, as the CRLB exceeded threshold value of<20%. Interestingly, we found significant decreases in GABA before BOLD-correction (p=0.048): however the line-broadening correction eliminated this trend, making it difficult to interpret whether the change originated from the experimental manipulation or merely reflected changes in line narrowing during the BOLD-signal. Future studies to quantify both excitatory and inhibitory neurotransmitters may need to use subtraction sequences such as MEGA-PRESS to obtain reliable identification of GABA signals during functional stimulation.

Our method addresses multiple technical challenges in the application of functional MRS. In previous studies, fMRI and fMRS were collected sequentially before (Mangia et al., 2006, Bednarik et al., 2015) or after (Just and Sonnay, 2017) the fMRS scan, or even in a different session (Mangia et al., 2007). Typically, BOLD-fMRI data were collected using shorter stimulation block-lengths that were significantly shorter than those used for fMRS (i.e. 30 s BOLD vs. 5.3 min fMRS, Bednarik et al., 2015; 10 s BOLD vs. 5 min fMRS, Schaller et al., 2013). Data collected at different time points make a direct comparison of BOLD-fMRI and MRS data less straightforward due to changes in physiological, cognitive and scanner-related variables between scans. Combined fMRI-MRS provides the closest link between hemodynamics and neurochemistry to date by allowing comparison on equal terms. Such simultaneity is critical in fMRS experiments, where the neurochemical response to the same stimulation can vary depending on stimulation period within prolonged stimulation blocks (Mangia et al., 2006, Bednarik et al., 2015) and even disappear upon repeats of the same stimulation (Michels et al., 2012). Comparability may become key for learning experiments, where one condition can only be learnt once; and the efficiency of obtaining two types of measures within the same time span may be vital in clinical populations and children, who are more prone to experience discomfort in the scanner.

Our experimental set-up employed a 7-Tesla scanner, in combination with the 32-channel coil and dielectric pad. This allowed us to achieve high signal-to-noise, permitting measurement of reliable changes in glutamate after only 64 s of visual stimulation, a time scale within recommended block-lengths for fMRI experiments (Amaro and Barker, 2006). Previous studies describing glutamate changes using block designs stimulated the visual cortex for 5 min and longer (Mangia et al., 2006, Lin et al., 2012, Schaller et al., 2013, Bednarik et al., 2015). However, it is well known that neural firing and accompanying change in hemodynamics adapt to repeated presentation (Boynton et al., 1996) and hemodynamic changes have been shown to cease after prolonged stimulation (Sonnay et al., 2015). Hence, prolonged stimulation paradigms are more susceptible to adaptation, signal drift, subject head motion, and decreases in attention and alertness. Future fMRS studies could take these design considerations into account, and aim for shorter block lengths, as presented in this study, to achieve maximal continuity with related functional measures. Other alternatives to maintain the amplitude of the BOLD-response include inserting short inter-stimulus-intervals between presentations (Sonnay et al., 2015), or using event-related presentation paradigms (Apsvalka et al., 2015). We have demonstrated that our scanning procedure allowed us to capture changes in glutamate at 64 s, a time scale suitable for functional MRI studies.

The T2* changes during the BOLD-response affect quantification of MR spectra through line narrowing (Zhu and Chen, 2001), which can bias metabolite quantification (Mangia et al., 2006). We have evaluated the BOLD-effect in MR-spectra by estimating line width changes on the tCr singlet, and then corrected for it by subject specific line broadening of spectra acquired during stimulation (Mangia et al., 2007, Bednarik et al., 2015). While glutamate changes were significant before and after BOLD-correction, other metabolite changes were not. We therefore rule out the possibility that changes in glutamate are caused by T2* -line narrowing during the BOLD-response.

One potential limitation of the methodology of this study is the use of a basis set in which multiplets of the simulated spectra of NAA and NAAG are separated. At moderate echo times, it has been shown that the singlet of NAA and NAAG became smaller due to the transverse relaxation, whereas the multiplet resonances of NAA not only became smaller due to the transverse relaxation but also underwent J-modulation (Deelchand et al., 2012, Marjanska et al., 2012). In this study, an additional freedom to LCModel analysis was introduced by splitting the singlets and multiplets of NAA and NAAG spectra in the basis set (Deelchand et al., 2012, Marjanska et al., 2012). Although this approach resulted in excellent fits, the exact quantification of peaks close to this range such as Gln and GSH might be affected. Another limitation of the methodology of this study is the semi-quantification of metabolite levels using unsuppressed water signal as an internal reference method within the same voxel, since the estimated changes were not corrected for the gray matter and white matter fractions within the voxel and metabolite T2s.

Glutamate exists in the brain in neuronal, glial, and metabolic pools with different turnover rates (Shank et al., 1993, Rae, 2014). MRS visibility of glutamate is determined by the biochemical context in which glutamate is present. Approximately 79% of glutamate in brain tissue is visible, with the visibility determined by the intracellular compartment (Kauppinen and Williams, 1991, Pirttila et al., 1993). Glucose consumption and blood flow increase during neural activity (Fox et al., 1988) and glutamatergic neurons account for up to 80% of glucose consumption in the resting human brain (Gruetter et al., 1998, Shen et al., 1999). Our observations are consistent with the view that glutamate plays a major role during activity-dependent energy demands in the cerebral cortex (Rothman et al., 2003), and specifically strengthen the link between BOLD-fMRI and glutamate during brief periods (64 s) of stimulation. In particular, we provide evidence supporting a relationship between BOLD-activation and glutamate during brief periods (64 s) of stimulation. Glutamate increases during visual stimulation could reflect increases in rate and magnitude of synaptic glutamate release during neuronal firing.

The ability to learn new skills and master them with practice is a process that requires dynamic adjustment of neuronal responses (Koralek et al., 2012), responses ultimately under control of glutamatergic signaling (Popoli et al., 2011). Functional changes during active learning could be investigated using the combined fMRI-MRS tool, providing a way to track changes in neurotransmitter concentrations while information is being consolidated. Such insight could be critical in helping patients adjust to neuroprosthetic devices and rehabilitate after limb or brain damage. Charting functional neurochemistry over lifespan could help to understand why children are better learners than adults. The glutamatergic system is also a mechanistically important player to understand cognitive disorders. Schizophrenia is a neuropsychiatric disorder that is thought to involve a network of cortical and subcortical areas with glutamatergic connections (Moghaddam, 2003). The combined fMRI-MRS tool is ideally poised to map the functional neurochemical relationships between areas. For example, fMRI-MRS could be used to measure neurochemical levels during different memory tasks in the prefrontal cortex or the hippocampus, while BOLD-fMRI could be used to monitor activity levels across the brain. Such complementary information could help relate functional impairments to disease progression.

The ultimate aim of neuroscience research is to understand how multiple complex neural events contribute to the overall functioning of the brain: simultaneous acquisition of complementary measures is essential to achieving this goal (Hall et al., 2016). Our combined fMRI-MRS method has the potential to address questions about cortical function in the living human brain that cannot be answered by separate application of either technique alone.

## Competing interests

The authors have no competing financial or non-financial interests.
