# Supplementary material for: Combined fMRI-MRS acquires simultaneous glutamate and BOLD-fMRI signals in the human brain
Source: Neuroimage. 2017 Jul 15;155:113–9. doi: 10.1016/j.neuroimage.2017.04.030 (PMC5519502; doi:10.1016/j.neuroimage.2017.04.030)
Supplement: Supplementary file 1 — Supplementary material [file mmc1.docx]

**SUPPLEMENTARY MATERIALS**

Figure S1: Magnetic Resonance Spectra for individual participants. Each spectrum is the average of 128 spectra. The x-axis shows frequency of chemical shift in ppm. Spectral heights have been normalized to the NAA-singlet peak at 2.01 ppm.

Figure S2: Panel shows single subject glutamate response superimposed onto the BOLD-fMRI response. White areas indicate baseline and gray area visual stimulation. Pale red section shows glutamate concentration in the first baseline period. Red lines show glutamate concentrations after the first baseline period. Black lines show z-normalized BOLD-change, which has been averaged across four consecutive data points.
